# FACdb: a comprehensive resource for genes, gut microbiota, and metabolites in farm animals

**DOI:** 10.3389/fmicb.2025.1557285

**Published:** 2025-03-21

**Authors:** Ze Zhang, Yang Li, Di Zhang, Shuai Chen, Sien Lu, Kang Wang, Miao Zhou, Zehe Song, Qingcui Li, Jie Yin, Xiaoping Liu

**Affiliations:** ^1^Key Laboratory of Systems Health Science of Zhejiang Province, School of Life Science, Hangzhou Institute for Advanced Study, University of Chinese Academy of Sciences, Hangzhou, China; ^2^BGI Research, Hangzhou, China; ^3^College of Animal Science and Technology, Hunan Agricultural University, Changsha, China; ^4^School of Pharmaceutical Science and Technology, Hangzhou Institute for Advanced Study, University of Chinese Academy of Sciences, Hangzhou, China

**Keywords:** farm animal, connectome database, gene expression, gut microbiota, metabolites, association networks

## Abstract

Farm animals, including livestock and poultry, play essential economic, social, and cultural roles and are indispensable in human welfare. Farm Animal Connectome database (FACdb) is a comprehensive resource that includes the association networks among gene expression, gut microbiota, and metabolites in farm animals. Although some databases present the relationship between gut microbes, metabolites, and gene expression, these databases are limited to human and mouse species, with limited data for farm animals. In this database, we calculate the associations and summarize the connections among gene expression, gut microbiota, and metabolites in farm animals using six correlation or distance calculation (including Pearson, Spearman, Cosine, Euclidean, Bray–Curtis, and Mahalanobis). FACdb contains over 55 million potential interactions of 73,571 genes, 11,046 gut microbiota, and 4,540 metabolites. It provides an easy-to-use interface for browsing and searching the association information. Additionally, FACdb offers interactive visualization tools to effectively investigate the relationship among the genes, gut microbiota, and metabolites in farm animals. Overall, FACdb is a valuable resource for understanding interactions among gut microbiota, metabolites, and gene expression. It contributes to the further utilization of microbes in animal products and welfare promotion. Compared to mice, pigs or other farm animals share more similarities with humans in molecular, cellular, and organ-level responses, indicating that our database may offer new insights into the relationship among gut microbiota, metabolites, and gene expression in humans.

## Highlights

- FACdb integrates gene expression, gut microbiota, and metabolite data for farm animals, filling a critical gap in current databases.
- Offers over 55 million interactions among 73,571 genes, 11,046 gut microbiota, and 4,540 metabolites.
- Features a user-friendly interface with tools for efficient browsing and visualization of complex associations.
- FACdb reveals molecular and cellular similarities between farm animals and humans, with applications in both agriculture and human health research.

## Introduction

Gut microbiota, which parasitizes the host’s intestinal tract, affects the intestinal barrier and immune function by producing various metabolites (Fan et al., 2021; De Vos et al., 2022; Willson et al., 2018; Zhang et al., 2016). These metabolites have a significant impact on intestinal homeostasis and host health. The relationship between gut microbiota and food digestion and absorption (Bergamaschi et al., 2020), immune system development (Li X. et al., 2022), growth, and development (Li et al., 2021) has been well established. The dysbiosis of gut microbiota can lead to the emergence and progression of enteritis, pancreatitis, and other diseases (Lavelle and Sokol, 2020; Lee et al., 2022; Zhu et al., 2019). Manipulating gut microbiota through exogenous factors is an imperative therapeutic intervention for the host diseases (Azimirad et al., 2020; Kanika et al., 2015; Wang et al., 2021). Therefore, comprehending the interaction mechanism between gut microbiota and hosts is imperative.

The metabolites of gut microbiota are diverse, including short-chain fatty acids, bile acids, and trimethylamine-*N*-oxide (TMAO) (Donia and Fischbach, 2015; Krautkramer et al., 2021). These molecules interact with various host cell receptors (De Vos et al., 2022; Yonezawa et al., 2007; Zheng et al., 2019), including Toll-like receptors (TLRs), G protein-coupled receptors (GPCRs), and endogenous cannabinoid receptors, to regulate the signal pathways and impact physiological functions (Bates et al., 2006; Hu et al., 2018), such as the intestinal barrier and secretion (Wang L. et al., 2023). The development of molecular tools and techniques such as macrogenomics, metabonomics, lipidomics, and macrotranscriptomics has enabled the gradual decoding of complex interactions between hosts and various microorganisms (Baker, 2023; Li L. -Y. et al., 2022; Pérez-Cobas et al., 2013; Walsh et al., 2017; Zhou et al., 2023). Several databases have been published to describe the target genes of gut microbiota and their microbial metabolites in humans and mice (Cheng et al., 2022; Wu et al., 2020). Understanding the connection between gut microbiota, their metabolites, and target genes establishes the basis for investigating the regulatory function of gut microbiota in the growth, development, and diseases of the host.

However, farm animals, which provide a plentiful source of meat, eggs, and dairy products for human and play an essential role in society, have been less studied in this context. Moreover, intestinal microbiota also impacts the animals’ ability to convert feed into nutrients and the quality of the meat and eggs (Wessels, 2022). Dietary interventions aimed at regulating these gut microbiota can potentially improve the overall health of farm animals and enhance the quality of agricultural and related products (Liao and Nyachoti, 2017). Dietary supplements may address animal illnesses by interfering with gut microbiota, facilitating nutrient absorption, and augmenting the nutritive value of meat and egg-based foods (Fathima et al., 2022). Despite the importance of these interactions, there remains a dearth of databases that incorporate the network of connections between gene expression, gut microbiota, and metabolites specifically in farm animals. This lack of comprehensive resources hinders our understanding of the impact of gut microbiota on farm animal health and productivity.

To address this gap, we constructed the Farm Animal Connectome database (FACdb). The primary objective of FACdb is to investigate the function of gut microbiota in farm animals by integrating genomics, intestinal microflora, and metabolic data. The novelty of FACdb lies in its comprehensive approach to mapping the associations between these biological layers—gene expression, gut microbiota, and metabolites—specifically in farm animals. Furthermore, the FACdb features a user-friendly interface that allows for easy information browsing and searchability, along with interactive visualization tools, thus making complex data more accessible and actionable. FACdb is publicly available without login requirements at http://compbiol.top:2023/FACdb/ or http://122.224.251.240:2023/FACdb/.

We hope this database will offer novel insights into the complex correlations between intestinal microflora, metabolites, and host gene expression, ultimately advancing research in farm animal health, productivity, and the broader fields of microbiome research.

## Materials and methods

### Data sources

For the construction of the FACdb database, a valuable collection of omics resources from various sources was obtained. These resources included literature sources (Chen et al., 2023; Liu et al., 2020; Mu et al., 2022) and manually collected data. The raw data collection focused on three essential types of data: gene expression, gut microbiota, and metabolites, to facilitate the construction of connectomes.

Each sample of each species had at least three types of omics data to investigate the interrelationships among the three types of datasets in farm animal species. Through the processing of multivariate data, cleansed data of four farm animal species were integrated into the database: *Sus scrofa* (pig), *Bos taurus* (cattle), *Ovis aries* (sheep), and *Gallus gallus* (chicken). The sample numbers for each species are provided in Table 1.

**Table 1:** The amount of samples in FACdb for each organism.

	*Sus scrofa*	*Bos taurus*	*Ovis aries*	*Gallus gallus*
Sample	34	23	12	35

Data integrationAs for gut microbiota data, quality control of the 16S sequence reads was performed using Trimmomatic (version 0.39) (Bolger et al., 2014) to discard adaptors and low-quality reads with the following criteria: bases were cut off the start or the end of a read if receiving a quality score of <3; Reads were truncated at any site receiving an average quality score of <20 over a 5-bp sliding window, discarding the truncated reads that were shorter than 36 bp. The remaining reads were further denoised into amplicon sequence variants (ASVs) using DADA2 (Callahan et al., 2016) in QIIME2 (Bolyen et al., 2019) according to the default parameters. Based on the Greengeens2 (version 2022.10) (McDonald et al., 2023), we got the final taxonomy matrix through a species comparison annotation using a q2-feature-classifier (Bokulich et al., 2018).

As for single-cell RNA sequencing (scRNA-seq) data, Trim Galore^1^ was used for quality control of raw data and junction trimming with the default parameters. Then, we mapped the raw data of each species to their reference genomes downloaded from the NCBI database^2^ and performed transcript quantification using Salmon (version 1.10.1), separately. The count gene expression matrix was normalized to transcripts per kilobase of exon model per million mapped reads (TPM).

### Data standardization

In order to ensure the compatibility and comparability of the integrated cleansed data in the FACdb database, a rigorous data standardization and normalization process was conducted. This step was essential to address discrepancies and variations across different studies and platforms and construct structured data for FACdb. Structured data refers to the aligned information of three types of omics data samples for each species, encompassing both the data related to each omics type and their associated attribute information.

#### Gene expression

The gene expression data was standardized by applying a log2 transformation to the expression values.


$$x^{\prime }=\log_{2}\left(x+1\right)$$

Specifically, x represents the original expression value. This transformation was employed to stabilize the variance and achieve a more symmetrical data distribution.

#### Gut microbiota

The gut microbiota data was normalized using column normalization.


$$x^{\prime }=\frac{x}{\sum x}$$

This normalization method involved calculating the relative abundance of each microbial taxon ( x ) by dividing the abundance of the taxon by the sum of abundances across all taxa in a sample ( ∑x ). This approach ensures that the relative abundance of each taxon is represented proportionally.

#### Metabolites

The cleansed metabolite data was standardized by applying a $\log_{2}\left(x\right)$ to the abundance values. Log2-transformed metabolite abundances were scaled using the Pareto scaling method.


$$x^{\prime }=\frac{x-\bar{x}}{\sqrt{s}}$$

Specifically, x represents the original abundance value. The Pareto scaling method involves dividing each log2-transformed abundance value by the square root of the standard deviation of that metabolite across all samples. This scaling approach helps to reduce the influence of metabolites with high variances.

### Data extension

After applying the above standardization and normalization techniques, the three types of omics data were, respectively, linked to external databases. Gene data were mapped to String-DB^3^ (Szklarczyk et al., 2021), gut microbiota was linked to taxon ID in NCBI Taxonomy,^4^ and metabolite data were mapped to HMDB^5^ (Wishart et al., 2022). Finally, three types of structured data were obtained, and the data quantities for each category are shown in Table 2.

**Table 2:** The amount of omics data in FACdb for each organism.

	*Sus scrofa*	*Bos taurus*	*Ovis aries*	*Gallus gallus*
Gene	15,606	16,030	14,257	12,309
Gut microbiota	652	98	401	9,895
Metabolite	751	217	1957	1,615

### Correlation calculation

The data from the structured dataset are calculated by using six different methods to construct connectomes of three types of biological components: genes, gut microbiota, and metabolites. The six methods are introduced as follows:

Pearson correlation coefficient (Pearson and Galton, 1997): Measures the linear relationship between two variables. It is used when the variables have a linear relationship and follow a normal distribution.

Spearman’s rank correlation coefficient (Spearman, 1904): Measures the rank correlation between two variables. This non-parametric method is useful for non-linear relationships or when data is not normally distributed.

Cosine similarity (Manning et al., 2008): Measures the similarity between two vectors based on the angle between them. It is effective for high-dimensional data where magnitude is less important than direction.

Euclidean distance (Aggarwal et al., 2001): Measures the straight-line distance between two points in a multidimensional space. It is widely used in clustering and classification tasks.

Bray–Curtis dissimilarity (Bray and Curtis, 1957): Measures the dissimilarity between two vectors. It is commonly used in ecology to compare the relative abundance of species or features.

Mahalanobis distance (Mahalanobis, 1936): Measures the distance between two vectors, accounting for correlations and covariance structure. It is useful for multivariate data and when variables are correlated.

And the six correlation or distance calculation methods are shown in Table 3.

**Table 3:** The calculation methods of constructing connectomes.

Correlation methods	Formula	Goal
Pearson correlation coefficient (Pearson and Galton, 1997)	$r_{xy}=\frac{\sum_{i=1}^{n}\left(x_{i}-\bar{x}\right)\left(y_{i}-\bar{y}\right)}{\sqrt{\sum_{i=1}^{n}{\left(x_{i}-\bar{x}\right)}^{2}}\sqrt{\sum_{i=1}^{n}{\left(y_{i}-\bar{y}\right)}^{2}}}$	Measures the linear relationship between two variables.
Spearman’s rank correlation coefficient (Spearman, 1904)	$r_{s}=\rho_{R\left(X\right),\;R\left(Y\right)}=\frac{cov\left(R\left(X\right),R\left(Y\right)\right)}{\sigma_{R\left(X\right)}\sigma_{R\left(Y\right)}}$	Measures the rank correlation between two variables.
Cosine similarity (Manning et al., 2008)	$\cos\left(\theta \right)=\frac{A\cdot B}{\left\|A\|\|B\right\|}=\frac{\sum_{i=1}^{n}A_{i}\times B_{i}}{\sqrt{\sum_{i=1}^{n}{\left(A_{i}\right)}^{2}}\times \sqrt{\sum_{i=1}^{n}{\left(B_{i}\right)}^{2}}}$	Measures the directional consistency or similarity between two vectors.
Euclidean distance (Aggarwal et al., 2001)	$d\left(x,y\right)=\sqrt{\overset{n}{\underset{i=1}{\sum }}{\left(x_{i}-y_{i}\right)}^{2}}$	Measures the Euclidean distance between two vectors.
Bray–Curtis dissimilarity (Bray and Curtis, 1957)	$D_{\mathrm{Bray}-\mathrm{Curtis}}=1-2\frac{\sum \min\left(S_{A,i},S_{B,i}\right)}{\sum S_{A,i}+\sum S_{B,i}}$	Measures the dissimilarity between two vectors.
Mahalanobis distance (Mahalanobis, 1936)	$Dis_{\mathrm{mahalanobis}}\left(x_{1},x_{2}\right)=\sqrt{{\left(x_{1}-x_{2}\right)}^{T}S^{-1}\left(x_{1}-x_{2}\right)}$	Measures the Mahalanobis distance between two vectors.

Since the structured data samples are aligned, the three types of biological entities are characterized as follows: genes by expression levels, gut microbiota by abundance values, and metabolites by peak area measurements within the samples.

To address the heterogeneity of different data types, the cleansed data undergo standardization or normalization to obtain structured data. These structured data are then subjected to preprocessing steps. Prior to the calculation of Euclidean and Mahalanobis distances, PCA (Hotelling, 1933) is applied to reduce dimensionality, mitigating the effects of the “curse of dimensionality” while preserving high-variance samples to capture differential relationships within the network. Subsequently, three types of interactions, namely, gene-gut microbiota, gene-metabolite, and gut microbiota-metabolite, constructed the connectivity network in FACdb, encompassing the relationships among the four species.

### Database content

**Figure 1:**
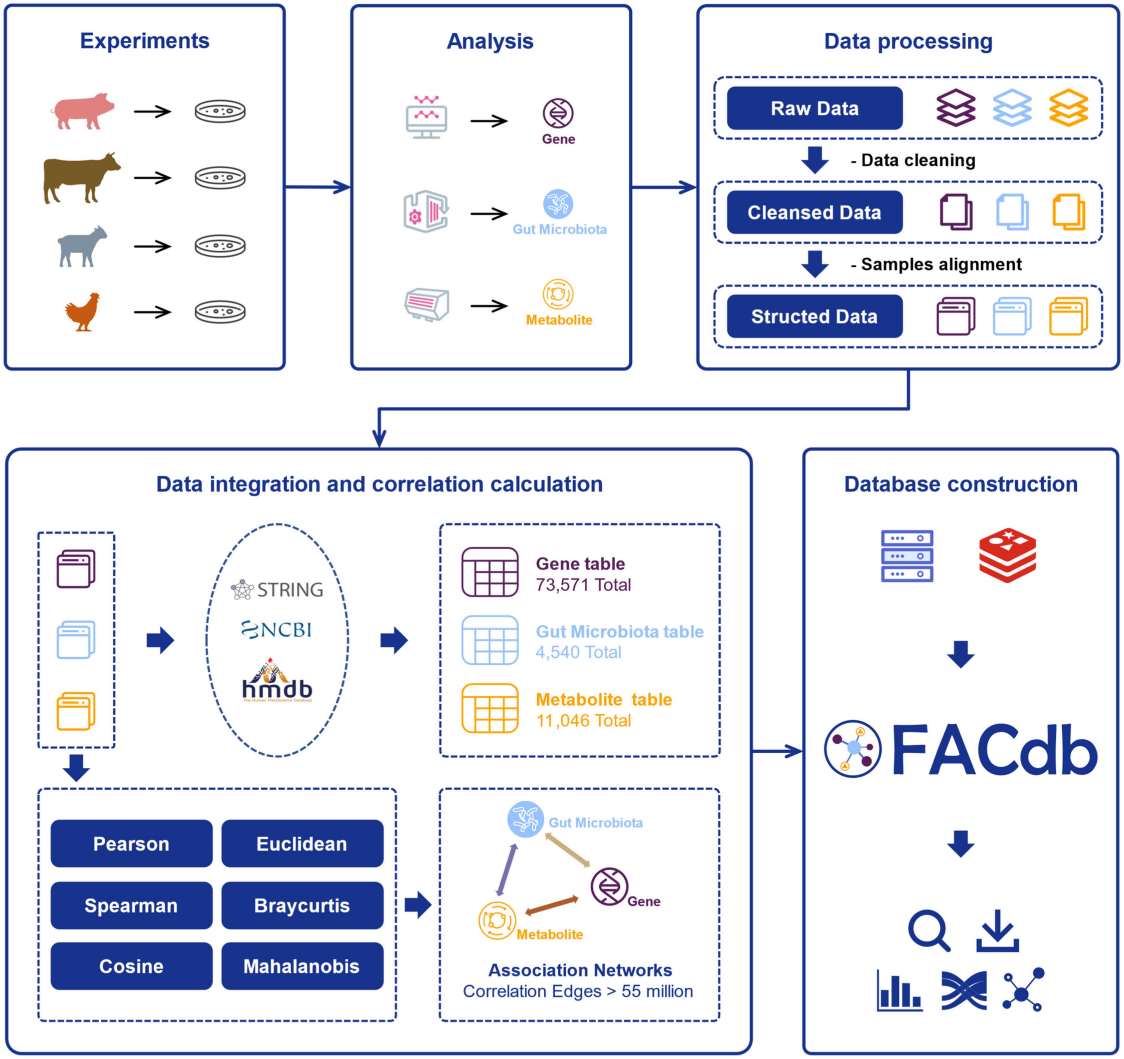
The flow chart of FACdb. Various samples from multiple organisms (pigs, cattle, sheep, and chickens) data were collected from public literature retrieval and experiments. The raw data for each organism, including gene expression, gut microbiota and metabolites, undergo processing and analysis. The raw omics data were cleaned, aligned, and processed to obtain structured data. And the structured data was integrated and subjected to correlation calculations to form interconnected networks. Finally, the FACdb web interface was constructed based on the Redis database, providing users with interactive data visualization.

After literature retrieval and experimental data collection, the multi-omics data of genes, gut microbiota, and metabolites from four species, namely pigs, cattle, sheep, and chickens, were obtained. These omics data were subjected to data cleansing, yielding cleansed data. Subsequently, structured data were obtained through sample alignment operations, ensuring alignment across the different types of data (Figure 1).

During the data integration process, on the one hand, we linked the structured data from different omics to corresponding data in external databases, enriching the information content. On the other hand, we employed six different computational methods to construct correlation networks among the three omics. In summary, FACdb encompasses a vast collection of potential interactions, comprising more than 55 million associations involving 73,571 genes, 11,046 gut microbiota, and 4,540 metabolites. It offers an intuitive interface that facilitates browsing and searching for information associated with associations. Moreover, FACdb provides interactive visualization tools to enable efficient exploration of the intricate relationships among genes, gut microbiota, and metabolites in farm animals.

Based on the integrated data, we have successfully constructed and deployed the FACdb (Farm Animal Connectome database), which provides a comprehensive database tool with convenient search functionality and interactive visualization. FACdb allows users to efficiently explore and analyze the complex relationships among genes, gut microbiota, and metabolites.

### Database construction and deployment

FACdb was constructed using a modern decoupled architecture with front-end and back-end separation. The front-end was built using Vite 4.0.0,^6^ Vue.js 3.0.3,^7^ TypeScript 4.8.4^8^, Element Plus 2.3.0,^9^ and Font Awesome 6.3.0^10^ to create a responsive and interactive user interface. The back-end was developed with Express 4.16.1,^11^ Node.js 18.14.2,^12^ and JavaScript to handle server-side logic and data access. To enable fast queries and data retrieval, FACdb stores all integrated multi-omics data directly in a Redis 5.0.7^13^ in-memory database. Redis provides rapid caching and lookup of the large association networks and underlying gene, gut microbiota, and metabolite data. Containerization technologies were leveraged by deploying the database within Docker^14^ containers for simplified distribution and deployment. The decoupled architecture, Redis caching, and containerization allow FACdb to serve interactive exploration and analysis of large-scale farm animal multi-omics networks through an intuitive web interface. The database is currently deployed on Linux on x86_64 (Ubuntu Server 20.04.2 LTS) and is publicly accessible without login requirements.

## Results

### Overview of web interface and functions

To ensure that users can use the FACdb datasets, an online website was developed to browse and query the information. The website is divided into Home, Browse, Search, Statistics, Download, and About.

#### Browse

**Figure 2:**
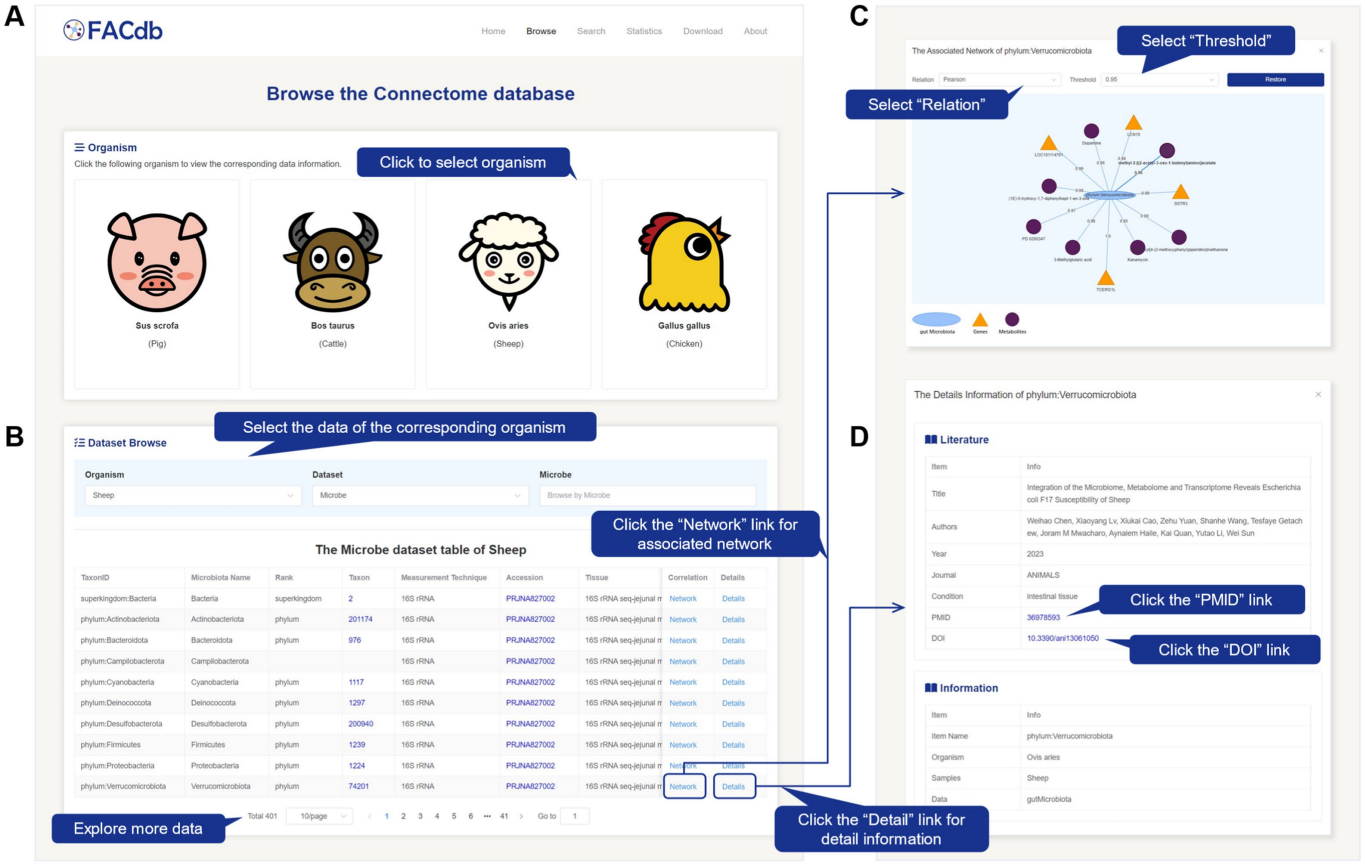
Screenshot of Browse page from FACdb. **(A,B)** Browse page. Users can select species cards. The retrieval results table is displayed below, with options to filter by “Species,” “Data Type,” and corresponding data name. **(C)** Pop-up window for data connection network corresponding to “Network” upon clicking, allowing users to filter network edges based on “Correlation Relationship Type” and “Threshold”. **(D)** Pop-up window for detailed information corresponding to “Detail” upon clicking.

On the Browse page, users can explore the basic information of gene expression, gut microbiota, and metabolite data (Figures 2A,B). By clicking the buttons under “Organism,” users can directly view data related to a specific species (Figure 2A). These details are displayed in the “Dataset Browse” section. Specifically, users can also perform targeted searches for gene, gut microbiota, or metabolite data of a particular species by selecting the species, dataset type, and specific molecular item. The retrieved data information table will be displayed at the bottom of the “Dataset Browse” section (Figure 2B). The gene information includes ID, Organism, Samples, Data, Gene Name, STRING protein ID, Throughput, Accession, Title, Year, Journal, Authors, Condition, DOI, and PMID. The gut microbiota information includes ID, Organism, Samples, Data, TaxonID, Gut Microbiota, Taxon, Rank, Measurement Technique, Tissue, Accession, Title, Year, Journal, Authors, Condition, DOI, and PMID. The metabolite information includes ID, Organism, Samples, Data, Metabolite, HMDB ID, Measurement Technique, Title, Year, Journal, Authors, Condition, DOI, and PMID.

By clicking the “Network” link, users can view a network visualization in a pop-up window. This network revolves around the selected item as the central node and displays the direct associations between the three types of items (gene, gut microbiota, and metabolite data) based on a specific correlation method and threshold condition. Genes are represented by orange-yellow triangles, gut microbiota by light blue ellipses, and metabolites by deep purple circles. The edge values between items indicate the association weights (correlation or distance). Users can further restore the network by selecting the desired “Relation” and corresponding “Threshold” from the dropdown menu in the pop-up window (Figure 2C).

By clicking the “Details” link, users can view further detailed information about the selected item in a pop-up window (Figure 2D). This information includes essential details such as literature sources and data types. Users can utilize the PMID link to access the corresponding literature in the PubMed database.^15^

**Figure 3:**
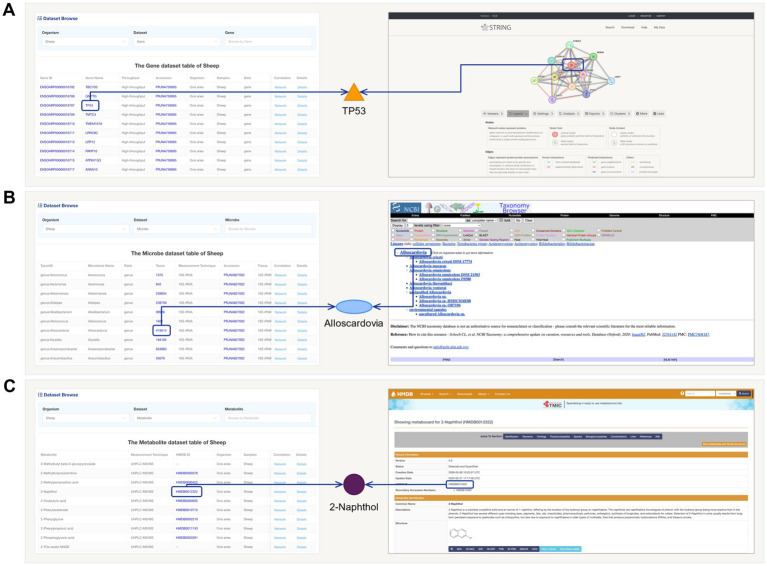
The schematic diagram of links and external database extensions in FACdb. **(A)** Conceptual diagram illustrating the association of “Gene Names” from FACdb gene data with “protein ID” from the STRING-DB. **(B)** Conceptual diagram illustrating the association of “Taxon” from FACdb gut microbiota data with NCBI Taxonomy. **(C)** Conceptual diagram illustrating the association of “HMDB ID” from FACdb metabolite data with HMDB.

For different data table information, we have provided targeted external database extensions. For example, in the Gene information data table, clicking on the “Gene ID” will redirect users to the PPI network of that gene in the STRING database (Figure 3A). Clicking on the “Gene Name” will lead to gene-related information in the NCBI database. Clicking “Accession” will allow users to access the original data and project information. Similarly, in the gut microbiota information data table, clicking on “Taxon” will trace the Taxonomy Browser information of that microorganism (Figure 3B). In the metabolite information data table, clicking on “HMDB ID” will provide access to the structure and other information of that metabolite in the HMDB database (Figure 3C).

**Figure 4:**
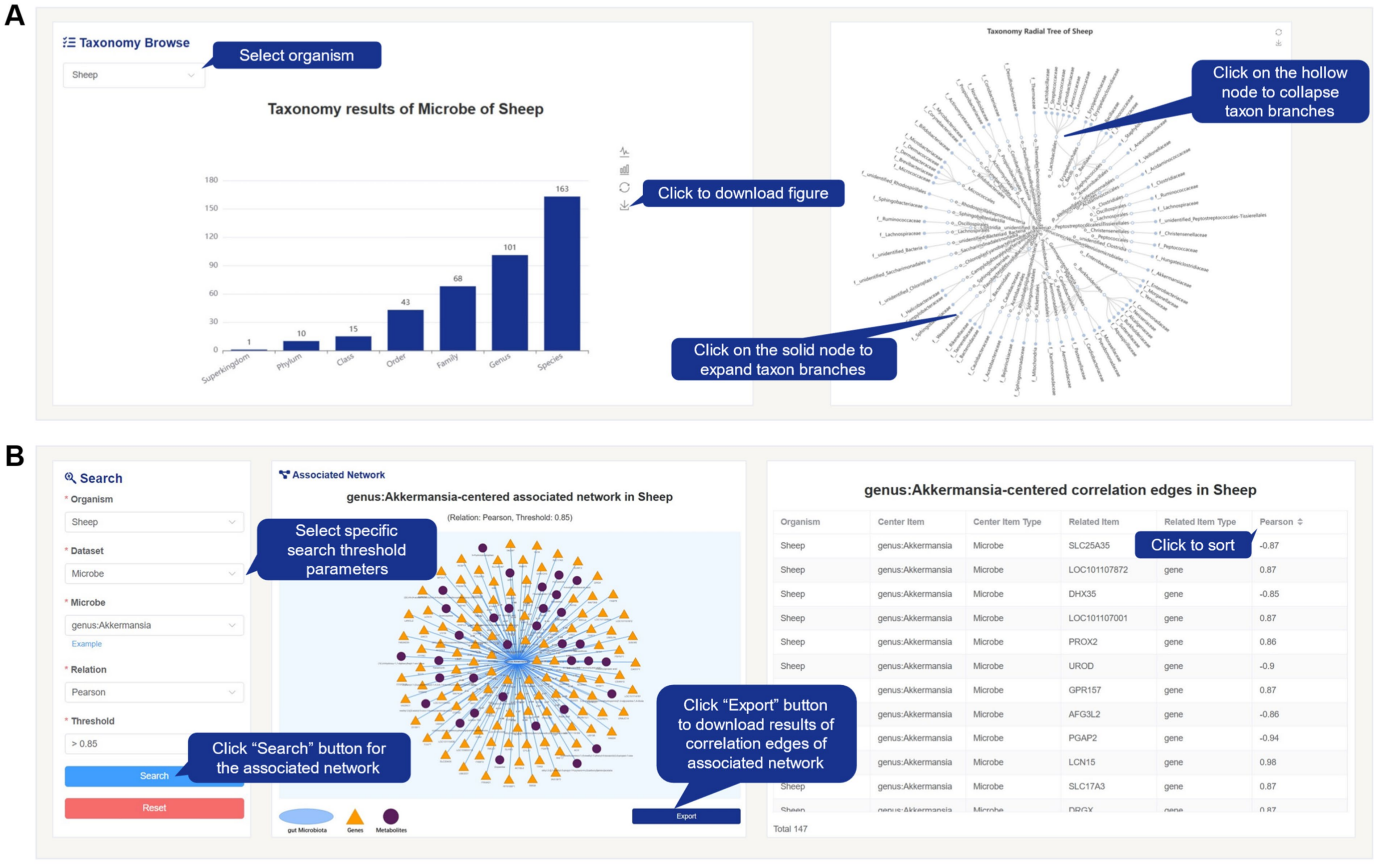
Screenshot of taxonomy results and Search page from FACdb. **(A)** Taxonomy results of gut microbiota in FACdb. FACdb provides interactive visualizations for gut microbiota data, including a bar chart displaying counts of “Superkingdom, Phylum, Class, Order, Family, Genus, Species” and a radial tree graph depicting hierarchical relationships. **(B)** Search page. Users can precisely retrieve data connection network information based on details like “Organism,” “Dataset,” “Relation,” and “Threshold”. Example queries are also provided. The visualization of the connection network and its edge information is displayed on the right. Users can click the Export button to download corresponding network and edge information.

Within the content of the Browse page, the microbial data for each species is matched with its taxonomy. The data is classified and summarized based on the hierarchical classification of “Kingdom, Phylum, Class, Order, Family, Genus, and Species.” The taxonomy tree graph displays the quantity information of taxonomy matches, and the radial tree graph represents the corresponding hierarchical relationships (Figure 4A). Users can interactively explore the visualized results by selecting the desired species from the “Species” dropdown menu. Additionally, users have the option to download the image results. Users can click on specific nodes in the radial tree graph to expand or collapse detailed information.

#### Search

The search page in FACdb offers users a more precise search method and provides detailed information of connectivity network data. Users can input their search criteria in the search input box by selecting “Organism” and “Dataset” and entering the specific item name. They can also set the “Relation” and “Threshold” values to retrieve highly targeted networks within FACdb. To assist users, we have provided category-specific examples within the search input box for reference (Figure 4B). Unlike the Browse page, the Search page also provides information on the edge weights of the networks. Users can interact with the page to sort and view the networks information based on the edge weights or export and download them for further analysis.

#### Statistics, download, and about

**Figure 5:**
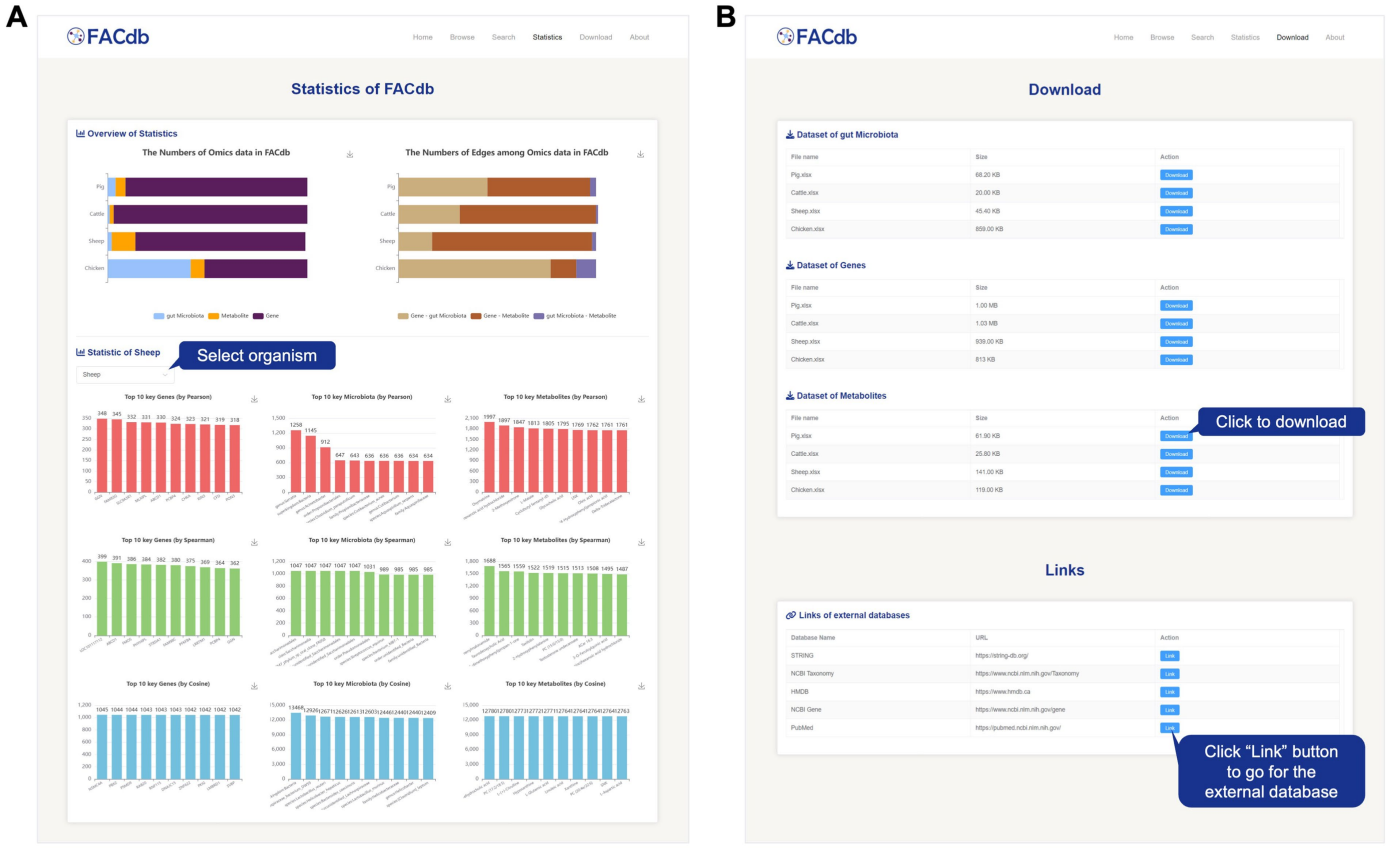
Screenshot of Statistics and Download page from FACdb. **(A)** Screenshot of statistics page from the FACdb. **(B)** Screenshot of download page from the FACdb.

The Statistics page presents the aggregated data in FACdb using visual charts. It includes an overview of statistics on the three types of omics data in FACdb and the connectivity network edges. Additionally, this page showcases the top 10 items for each type of data in terms of the number of edges in different species connectivity networks under three different correlation drivers (Figure 5A).

All data in FACdb are shared under the knowledge sharing license agreement, and we provide a download interface for users to download the data from FACdb and go for the extended databases (Figure 5B). To facilitate users to understand and use our database, we have developed an “About” page to query the explanations meaning of specific items in FACdb and the detail information of our team.

### Statistics analysis on database contents

In FACdb, we conducted a comprehensive statistical analysis to examine the quantity and associations between three types of items within each species, including genes, gut microbiota, and metabolites.

#### Distribution and classification of data in FACdb

**Figure 6:**
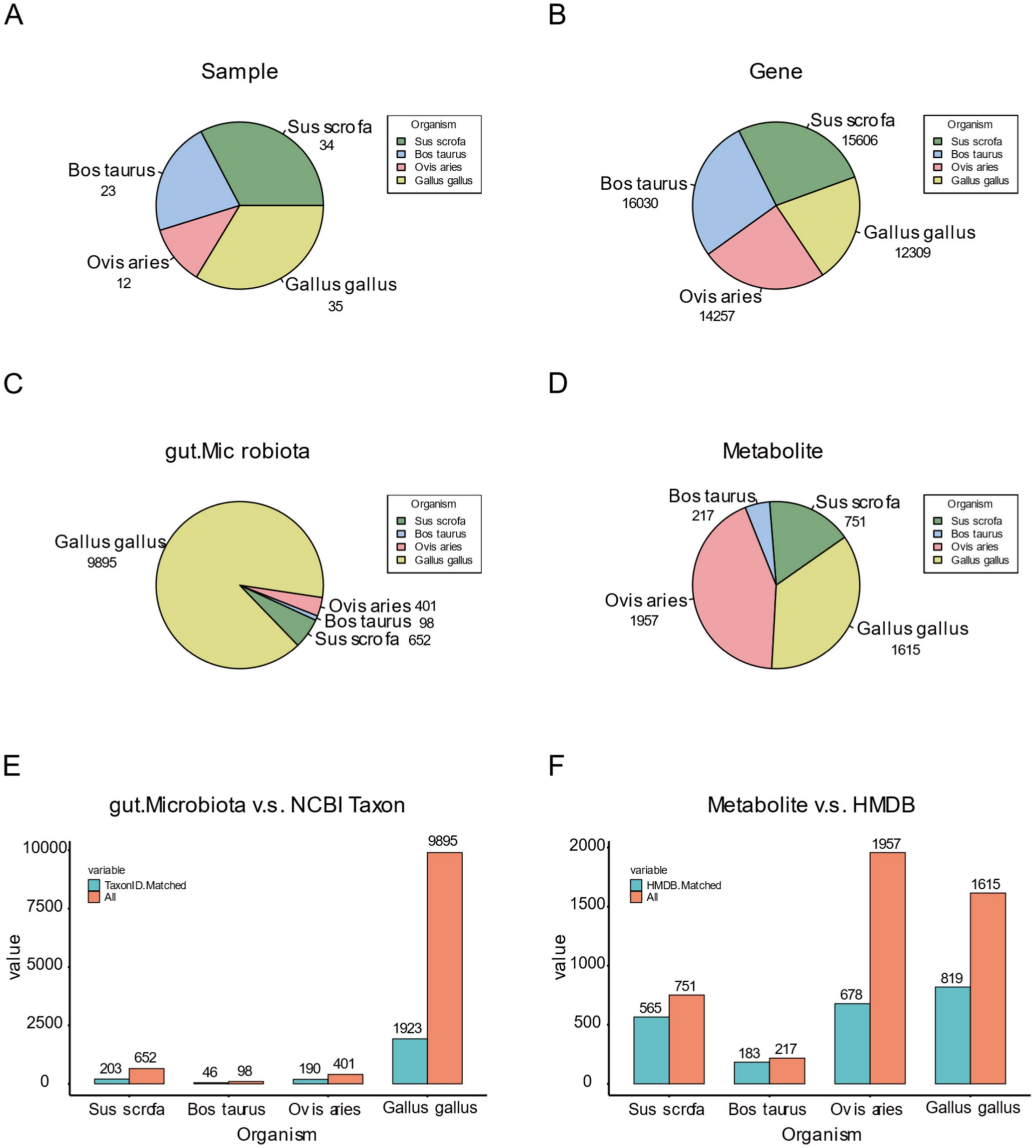
Data distribution plot of FACdb. **(A)** Pie chart illustrating the distribution of sample counts for four species in the FACdb. **(B–D)** Pie charts depicting the distribution of genes, gut microbiota, and metabolites counts for four species in the FACdb. **(E)** Bar chart displaying the distribution of gut microbiota matched with NCBI Taxonomy data across four species. **(F)** Bar chart presenting the distribution of metabolites matched with HMDB data across four species.

We conducted a comprehensive statistical analysis of sample distribution and the corresponding distribution of each omics data within each species in the FACdb database. To ensure the successful construction of interconnected components in FACdb, we applied a series of standardization processes and filtering operations. Samples with data from all three omics types (genes, gut microbiota, and metabolites) were selected based on stringent criteria, and the distribution of samples meeting the data conditions and exhibiting high quality were illustrated in Figure 6A, with 34 samples for pigs, 23 for cattle, 12 for sheep, and 35 for chickens (Figure 6A).

Furthermore, a pie chart was generated to visually represent the distribution of three omics data types among different species in FACdb. The distribution of genes is notably uniform across the four species (Figure 6B), indicating a relatively stable and conservative genetic evolution among farm animals in the transcriptional sequencing of genes. In contrast, gut microbiota distribution displays significant variations. Specifically, the gut microbiota data for pigs (652), cattle (98), and sheep (401) show minimal differences, while chickens (9895) exhibit a distinct lead (Figure 6C). This disparity can be attributed to the use of 16S sequencing for microbiota analysis in pigs, cattle, and sheep, and metagenomic sequencing for chickens, enriching the diversity of gut microbiota data in FACdb. The distribution of metabolite data, in comparison, shows moderate differences, with fewer entries for pigs (751) and cattle (217) and more for sheep (1957) and chickens (1615) (Figure 6D).

Additionally, for the scalability assessment of the FACdb database, we matched gene, gut microbiota, and metabolite data with external databases, including String-DB, NCBI Taxonomy, and HMDB (see Figure 3 for matching details). Due to minimal differences in genes, Figures 2E,F primarily showcase the distribution of gut microbiota and metabolite data matched with external databases. Figure 6E compares gut microbiota data for the four species in FACdb with NCBI Taxonomy. The substantial reduction in discrepancies caused by sequencing methods is evident, particularly in chickens, where the matched count in NCBI is 1923 out of 9,895 (Figure 6E). Figure 6F demonstrates the comparison of metabolite data for the four species in FACdb with HMDB, emphasizing the matching distribution between the metabolite of these family animals and human metabolite. Overall, the matching results for the metabolite of these four species to the human metabolite are relatively stable: pig (565/751), cattle (183/217), sheep (678/1957), and chicken (819/1615) (Figure 6F).

#### Pan-statistical analysis of data in FACdb

**Figure 7:**
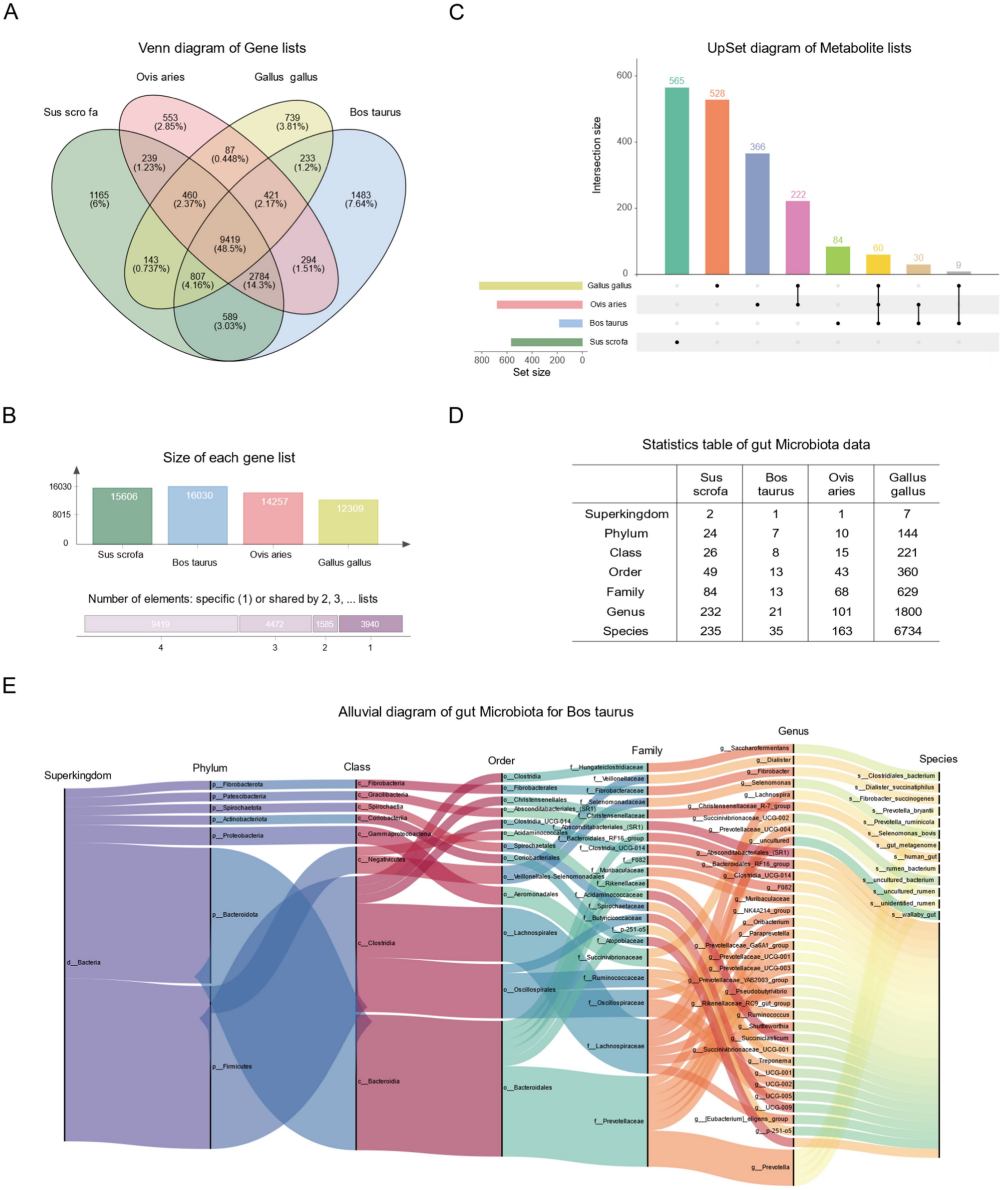
Statistical analysis plot of FACdb. **(A)** Venn diagram depicting the quantity of shared genes across different species in FACdb. Different colored circles represent data from distinct species. **(B)** The upper panel illustrates the gene count from samples of four species. The lower panel shows the gene count shared among samples belonging to 1–4 different species. **(C)** UpSet plot revealing the quantity of shared metabolites across different species in FACdb. **(D)** Comprehensive summary statistics of the counts of gut microbiota data from different species corresponding to “Superkingdom, phylum, class, order, family, genus, and species” categories in FACdb. **(E)** Alluvial diagram illustrating the hierarchical relationships of gut microbiota categories in cattle from FACdb.

Building upon the existing data, we conducted a personalized analysis of genes, gut microbiota, and metabolites in FACdb. We performed a quantitative intersection analysis for the transcriptomic gene lists of the four species in FACdb and visualized the results using a Venn diagram. The analysis revealed that 48.5% of genes, totaling 9,419, are shared among the four species (Figure 7A). Furthermore, approximately 80% of genes exist in common among these species, as calculated by $\left(9418+4472+1585\right)/\left(9418+4472+1585+3940\right)$ (Figure 7B), indicating a certain level of genetic conservation in the evolutionary perspective of these family animals. Similarly, Figure 7C illustrates the quantitative intersection analysis of metabolite compound names among the four species, accompanied by an UpSet plot (Figure 7C).

Concerning gut microbiota data, Figure 7D provides a detailed account of the number of taxonomy categories (Superkingdom, Phylum, Class, Order, Family, Genus, and Species) matched by gut microbiota in each family of animals in FACdb (Figure 7D). Additionally, Figure 7E presents a detailed Alluvial diagram showcasing the hierarchical relationships of gut microbiota data in cattle (Figure 7E). For enhanced user interaction, we have incorporated an interactive radial tree structure visualization of microbial taxonomic relationships on the website (refer to Figure 4A).

## Discussion

Genes, gut microbiota, and metabolites intricately weave a complex network of relationships (Durack and Lynch, 2018; Sung et al., 2017; Zhang et al., 2024; Zhang et al., 2023). Considering these interactions, we have built FACdb, focusing on family animals, to unravel the delicate connections between genes, gut microbiota, and metabolites. As for data in pigs, we queried gene-centric association networks in FACdb and conducted statistical analyses. For instance, we searched the *Ten-Eleven Translocation (TET)* gene family exhibited close associations with various gut microbiota and metabolites under the condition of Bray-Curtis <0.01 (details can be found in Supplementary Table S1 and Supplementary Figures S1A,B, S2–S4). Notably, the *TET* gene family (*TET1*, *TET2*, *TET3*) demonstrated proximity to *superkingdom: Bacteria*, *phylum: Bacteroidota*, *class: Bacteroidia*, and *order: Bacteroidales*. Further investigation, guided by previous studies, unveiled genes associated with antibiotic resistance in the pig gut microbiome, including *TET* genes encoding resistance to tetracycline antibiotics. These genes showed strong associations with bacterial genera recognized as cohabitants of the pig gut microbiome, such as *Bacteroides* spp. (Holman et al., 2017; Valeriano et al., 2017). These findings underscore the nuanced relationships among genes, gut microbiota, and more within biological processes as captured in FACdb. Similarly, in the case of sheep data, a statistical analysis of *genus: Akkermansia* data under conditions of three correlations (>0.85) and three distances (< 1) revealed a plethora of first-degree neighbor genes and metabolites (Supplementary Table S2; Supplementary Figure S1C). Previous research indicates that *Akkermansia Muciniphila*, a mucin-degrading symbiotic bacterium, is a promising probiotic candidate, potentially playing an indispensable role in metabolic activities within the mucosal layer (Zhang et al., 2019). These instances exemplify the wealth of intricate relationships embedded in the biological data of FACdb, shedding light on the interplay between genes, gut microbiota, and metabolites across different species.

Gut microbiota is intimately connected to host health, wellness, and growth (De Vos et al., 2022). Currently, the database for recording gut microbiota chiefly centers on the correlation between diverse intestinal microflora and varying physiological states of hosts or the impacts of disease interventions on gut microbiota (Cheng et al., 2020; Dai et al., 2022; Lei et al., 2023; Qi et al., 2023; Zhang et al., 2021). There are also reservations about the correlation between microbial genomes, metabolic groups, and human metabolites (Wishart et al., 2023). While there exists a database capturing the association between gut microbiota, microbial metabolites, and target genes in humans and mice (Cheng et al., 2022), research efforts in livestock have remained concentrated on the correlation between phenotype and gut microbiota (Xu et al., 2022). These farm animals boast considerable economic significance and provide humans with diverse meat, eggs, and dairy products. A comprehensive understanding of how gut microbiota in farm animals regulate the host is crucial. Our database addresses the research gap in the regulation of host target genes by gut microbiota in farm animals.

FACdb has significant potential to advance research in comparative genomics, gut microbiota studies, and veterinary sciences (Forcina et al., 2022; Wang C. -Y. et al., 2023). By integrating gut microbiota, metabolites, and host genes in farm animals, it enhances understanding of genetic and microbial interactions across species and aids in identifying regulatory mechanisms for health and disease (Dai et al., 2025; Furet et al., 2009; Ito et al., 2024). In microbiota research, FACdb provides insights into how microbiota regulate immune function, metabolism, and development, which is crucial for improving livestock health, productivity, and disease resistance. It also supports research into modulating microbiota through diet and probiotics. In veterinary sciences, FACdb links microbiota, metabolites, and host genes to identify biomarkers for diseases like enteritis and pancreatitis, enabling early diagnosis and personalized treatments. It also aids in studying the effects of microbiota-based therapies on host interactions. FACdb contains 73,571 genes, 11,046 intestinal flora, and 4,540 metabolites of four representative animal species. Six calculation methods were employed to construct an association network between these groups, yielding over 55 million associations. It facilitates researchers in establishing links between gut microbiota, their metabolites, and host target genes. However, the current database has limitations in sample numbers and animal species. Our next objective is to expand the database by incorporating more samples, species, and microorganisms, including viruses and archaea. The database we have established has facilitated research into the regulatory mechanisms of farm animal intestinal microbiota.

## Conclusion

We conducted extensive data collection and organization to provide information on genes, gut microbiota, and metabolites within the same samples across various farm animals, resulting in the development of FACdb. Utilizing raw data from published literature and experiments, we processed information from three omics dimensions, constructing association networks from diverse distances and correlation perspectives and aligning with external databases. FACdb web interface enables users to query and explore detailed information on the three omics and their association networks for each organism as needed. Interactive visualizations are provided, and all processed data are available for direct download on the website to enhance user-friendly access. FACdb will be continuously updated with new species data and high-quality omics information, serving as an interactive platform for family animal omics research. To our knowledge, FACdb is the first database incorporating multi-omics data, including genes, microbiota, and metabolites, across various farm animals, providing diverse association networks. In any case, FACdb is poised to be a robust tool for researchers exploring the biological significance of digestive metabolism, immune processes, and disease treatment by investigating family animal connectome networks.

## Data Availability

The original contributions presented in the study are included in the article/Supplementary material, further inquiries can be directed to the corresponding authors.

## Footnotes

^1^ https://github.com/FelixKrueger/TrimGalore

^2^ https://ftp.ncbi.nlm.nih.gov/genomes/refseq/

^3^ https://string-db.org/

^4^ https://www.ncbi.nlm.nih.gov/taxonomy/

^5^ https://hmdb.ca/

^6^ https://vitejs.dev/

^7^ https://vuejs.org/

^8^ https://www.typescriptlang.org/

^9^ https://element-plus.org/

^10^ https://fontawesome.com/

^11^ https://expressjs.com/

^12^ https://nodejs.org/

^13^ https://redis.com/

^14^ https://www.docker.com/

^15^ https://pubmed.ncbi.nlm.nih.gov/
