# Supplementary figures and images for: FACdb: a comprehensive resource for genes, gut microbiota, and metabolites in farm animals
Source: Front Microbiol. 2025 Mar 21;16:1557285. doi: 10.3389/fmicb.2025.1557285 (PMC11968756; doi:10.3389/fmicb.2025.1557285)

A

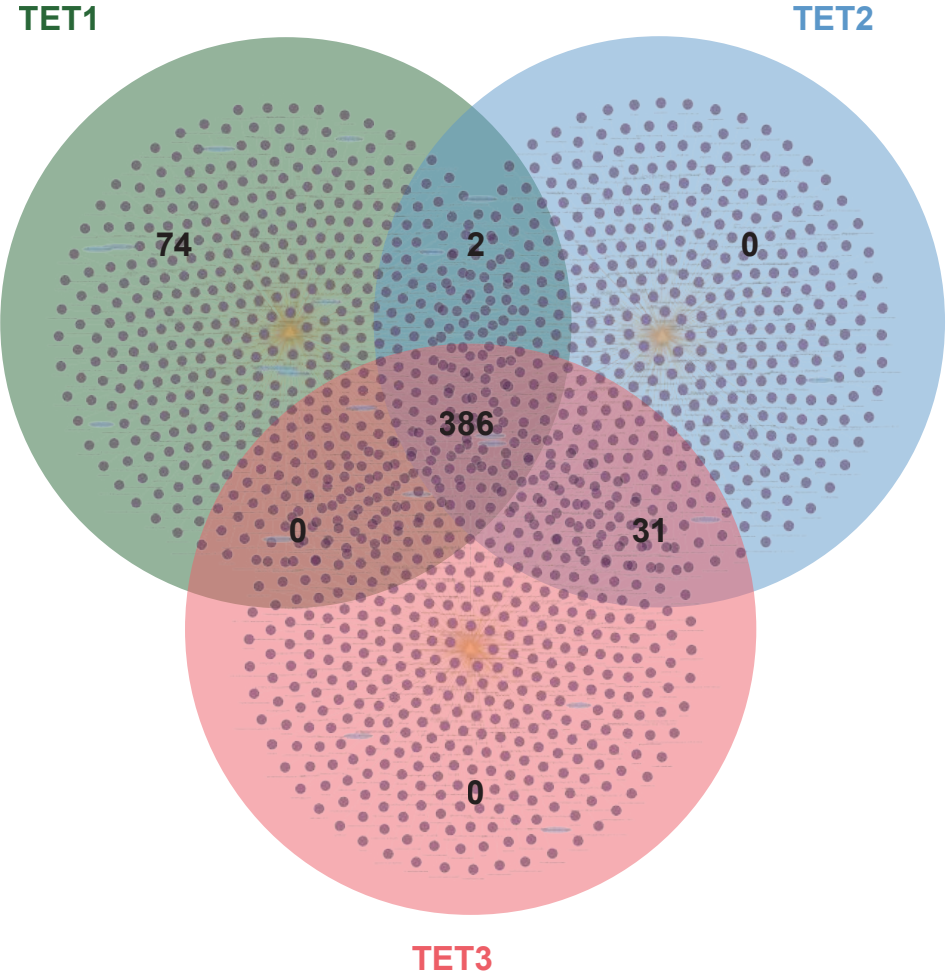

B

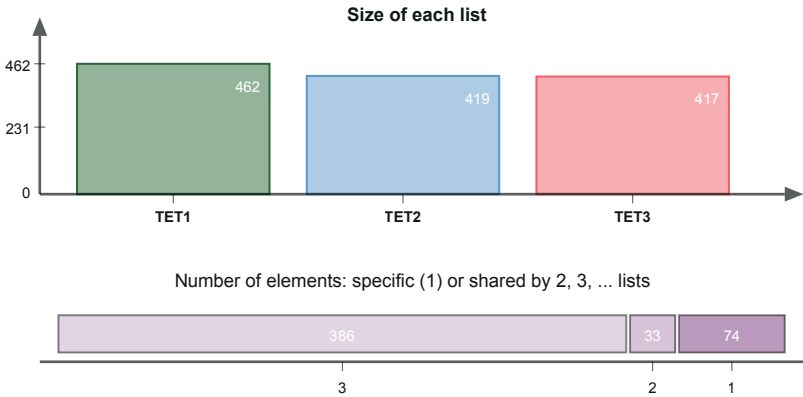

C

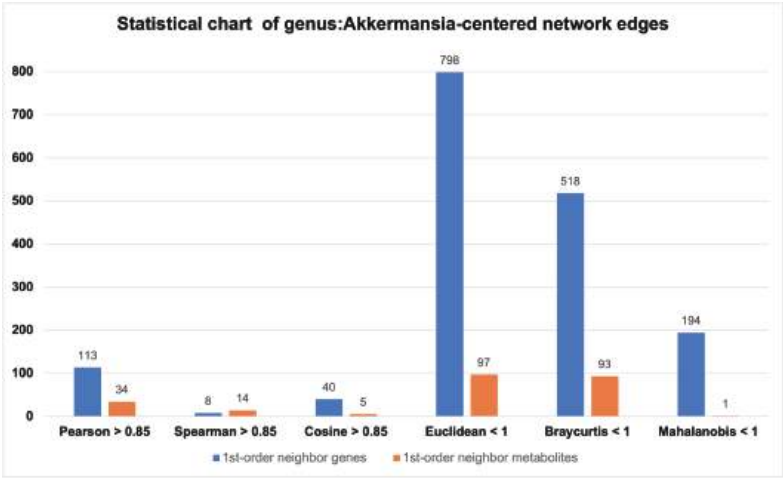

Supplement: Supplementary file 1 [file Data_Sheet_1.PDF]

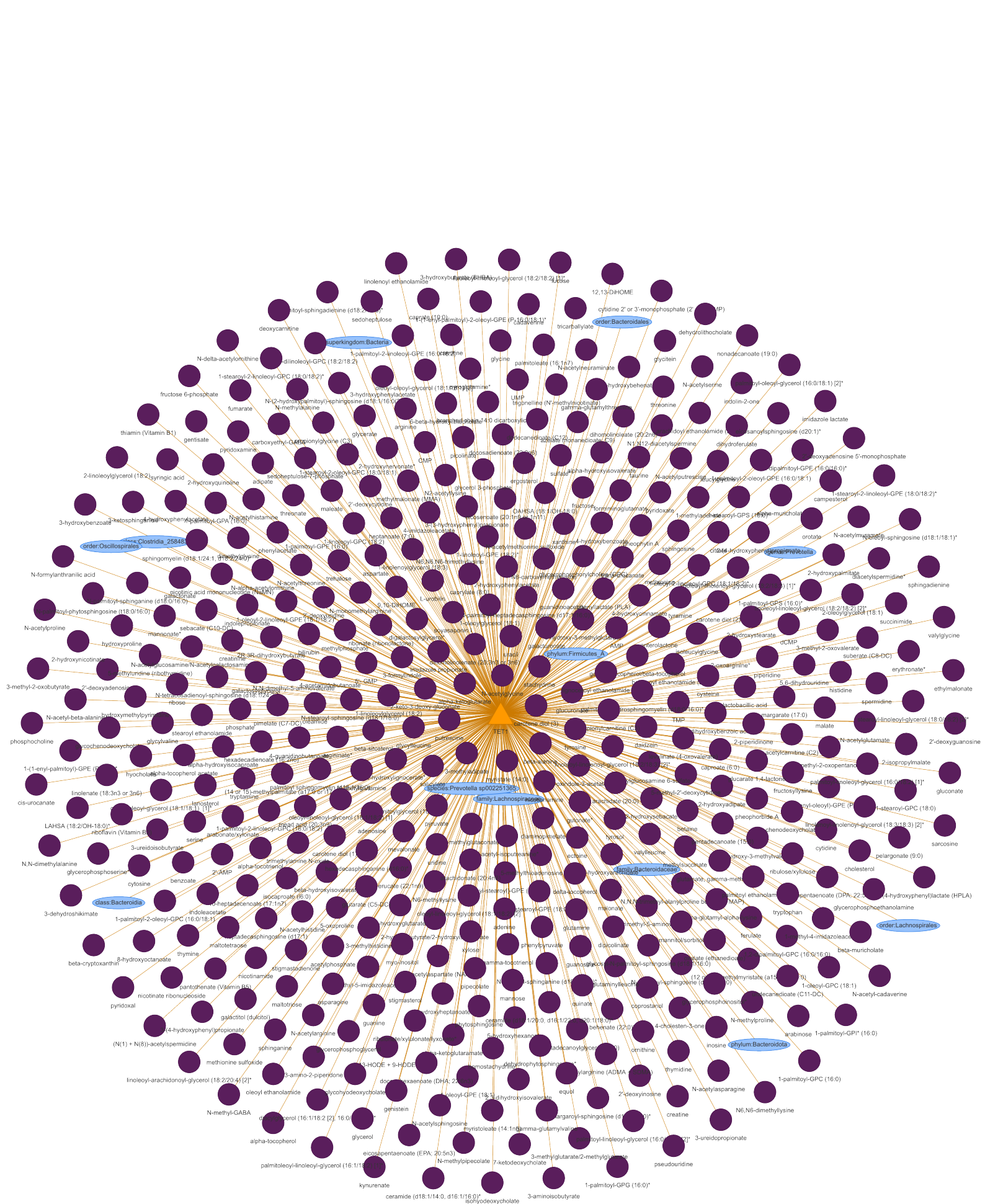

Supplement: Supplementary file 2 [file Data_Sheet_2.PDF]

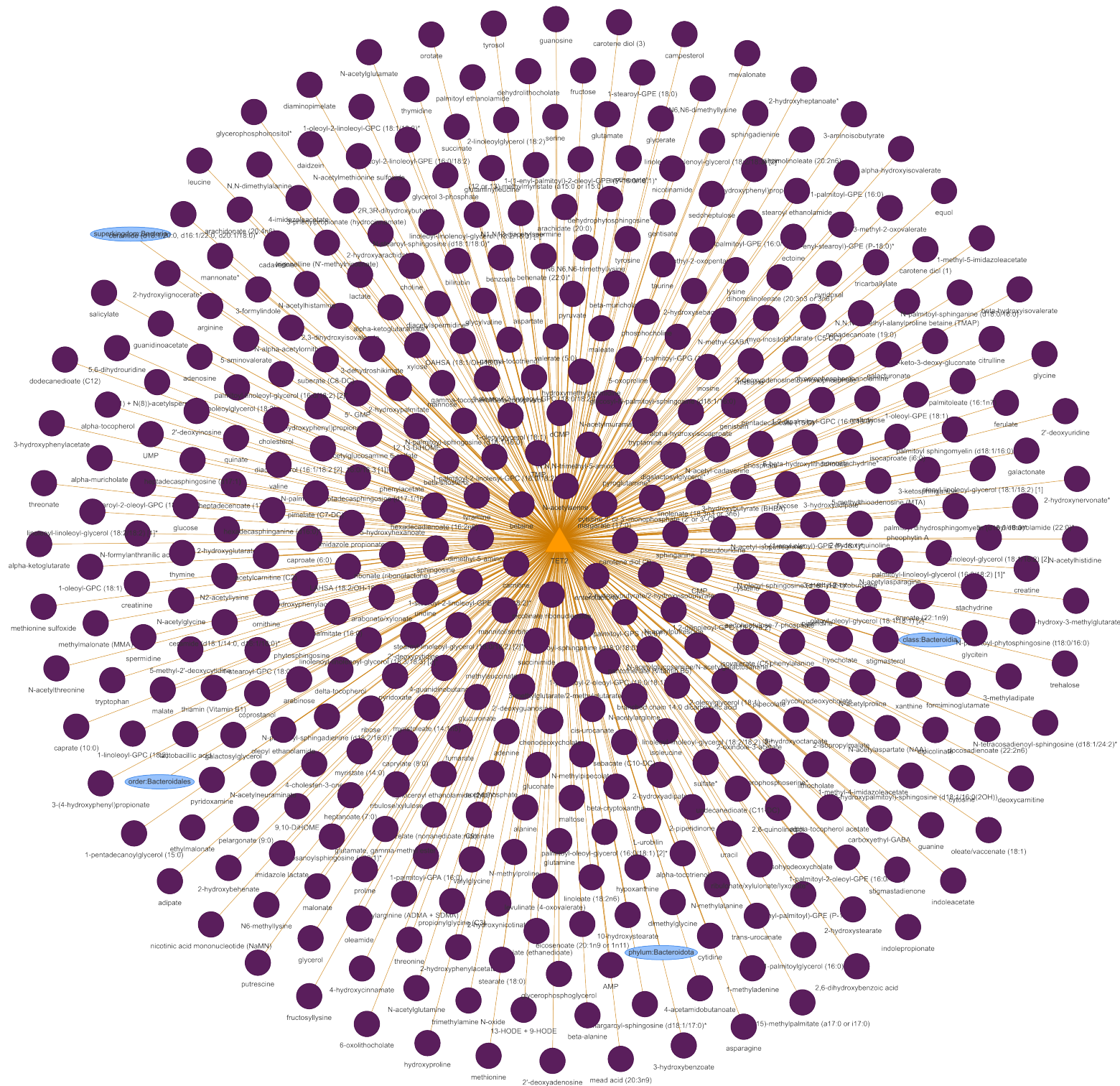

Supplement: Supplementary file 3 [file Data_Sheet_3.PDF]

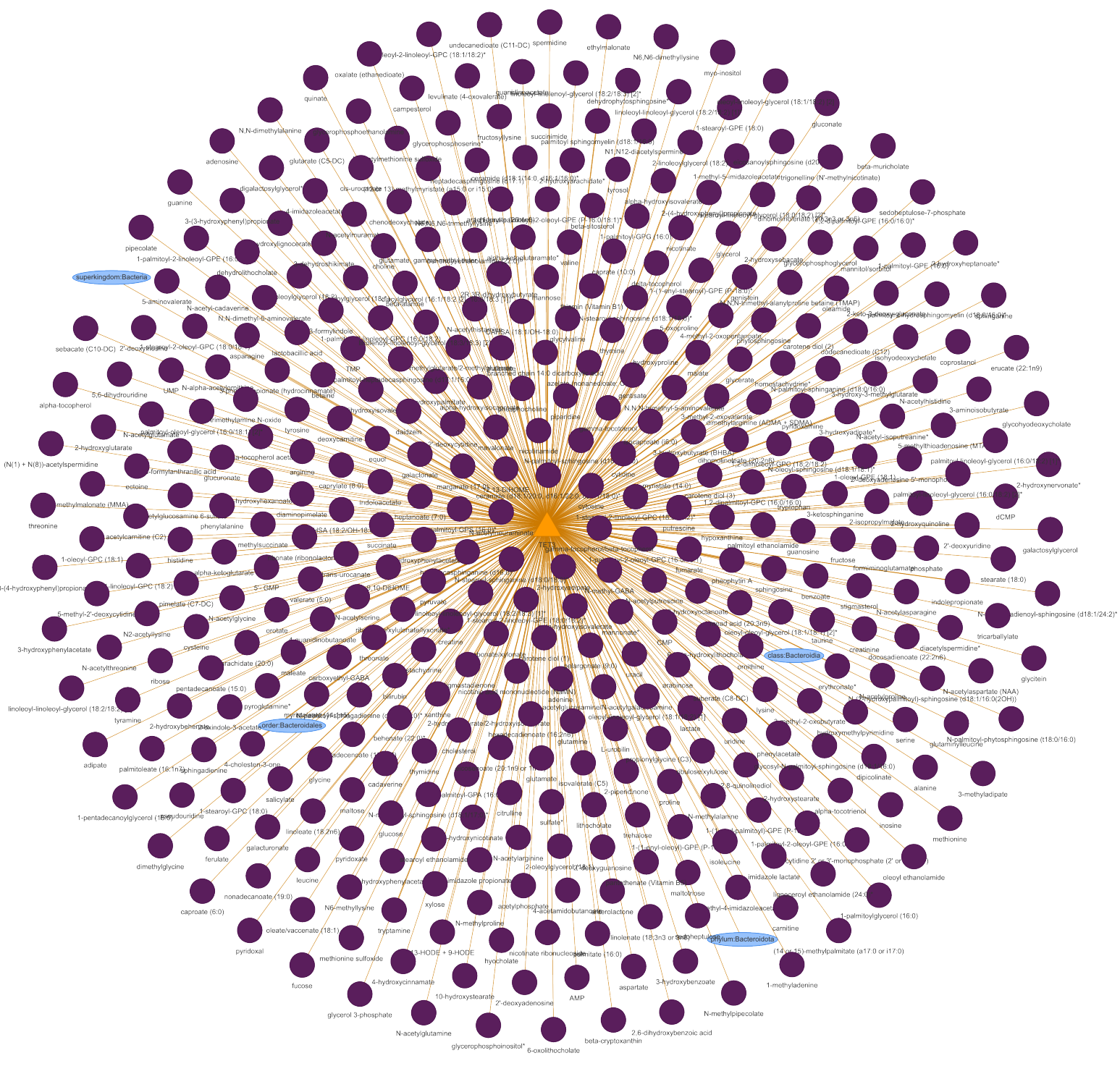

Supplement: Supplementary file 4 [file Data_Sheet_4.PDF]
